# Comment on “On the Utility of ToxCast™ and ToxPi as Methods for Identifying New Obesogens”

**DOI:** 10.1289/EHP881

**Published:** 2017-01-01

**Authors:** Keith A. Houck, Richard S. Judson, Thomas B. Knudsen, Matthew T. Martin, Ann M. Richard, Kevin M. Crofton, Anton Simeonov, Richard S. Paules, John R. Bucher, Russell S. Thomas

**Affiliations:** 1 National Center for Computational Toxicology, Office of Research and Development, U.S. Environmental Protection Agency, Research Triangle Park, North Carolina, USA; 2 National Center for Advancing Translational Sciences, National Institutes of Health, Department of Health and Human Services, Bethesda, Maryland, USA; 3 National Toxicology Program, National Institute of Environmental Health Sciences, National Institutes of Health, Department of Health and Human Services, Research Triangle Park, North Carolina, USA

Janesick and colleagues recently published an evaluation of the utility of ToxCast™ and Tox21 bioactivity data for predicting PPARγ activation and induction of adipogenesis. As providers of the ToxCast™ and Tox21 data as well as some of the chemicals employed in their follow-up study, we would like to comment on the methods Janesick and colleagues used in their application and interpretation of the data with respect to: *1*) incorrect ToxCast™/Tox21 citations in the main text of the article; *2*) lack of consideration of methodological, platform, and reagent differences when comparing the performance of individual ToxCast™ and Tox21 assays with their targeted studies; *3*) inconsistencies in some of the results reported by Janesick and colleagues on an individual assay basis; *4*) conclusions on the relative selectivity of the RXR-active chemicals; *5*) lack of consideration of technical and statistical factors; and *6*) incorrect integration of corollary data.

Through the ToxCast™ and Tox21 programs, the U.S. Environmental Protection Agency (EPA), National Toxicology Program (NTP), and National Center for Advancing Translational Sciences (NCATS) are committed to providing free and public data to support predictive toxicology efforts aimed at evaluating the potential hazard of environmental chemicals and facilitating scientific dialogue around the approach. We note that many of the data from ToxCast™ and Tox21 have been published in peer-reviewed journals, including the assay systems used in the study by Janesick et al. This includes, with correct citations, the NovaScreen cell-free biochemical assay platform (Knudsen et al. 2011; Sipes et al. 2013), the Attagene multiplex reporter gene assay platform (Martin et al. 2010), and the Tox21 reporter gene assays (Huang et al. 2011). The raw and processed ToxCast™ and Tox21 data and the computer code used to analyze the data can be downloaded from our website (https://www.epa.gov/chemical-research/toxicity-forecaster-toxcasttm-data). The processed data can also be accessed using the iCSS ToxCast™ Dashboard (https://actor.epa.gov/dashboard/). In addition, lessons learned from ToxCast™ Phase I have been thoroughly reviewed (Kavlock et al. 2012) in setting the path for ToxCast™ Phase II.

The analysis performed by Janesick and colleagues focused heavily on comparing the results from several ToxCast™ and Tox21 assays with different assays employed in their study (GAL4-mPPARγ transient transactivation assay in COS7 cells, and adipogenesis assays in 3T3-L1 cells and mBMSC cells). However, the authors failed to note a broad range of methodological and reagent differences that potentially confound a direct comparison of the results. One difference is that the ToxCast™ and Tox21 assays were human based, while those used by Janesick and colleagues were based on mouse or simian cells and the mouse PPARγ receptor. In addition, the ToxCast™ Attagene reporter gene assays use a human liver HepG2 cell line, variant HG19, with enhanced cytochrome P450 activity that can provide substantially different biotransformation capability than the cells used in their study. Enhanced CYP-mediated metabolism could also indirectly activate PPARγ through generation of reactive oxygen species and electrophilic metabolites (Bondy and Naderi 1994), leading to membrane oxidation and generation of bioactive lipids (Traber and Atkinson 2007) or induction of PPARγ coactivator PGC-1α expression (Wenz 2013). Supporting this possibility, many of the chemicals labeled as “active” in the ToxCast™ Attagene PPARγ assays also showed concordant NRF2 activity.

Another difference between Janesick et al. and the ToxCast™ and Tox21 results is the source of the test chemicals. The authors stated that the chemicals were “supplied by the National Toxicology Program (NTP) from the same stocks that were utilized in ToxCast™ Phase I.” Later in the paper the authors claimed that “[f]or analysis of the ToxPi, all chemicals tested were supplied by NTP.” Based on internal EPA and NTP records, both statements are incorrect. A total of 20 of the 21 chemicals listed in Table 1 and 5 of the 24 chemicals in Janesick et al.’s Table 2 were supplied to the authors by the EPA, while 10 of the same 20 chemicals supplied by the EPA in their Table 1 and all 5 of the chemicals supplied by the EPA in their Table 2 were also supplied by NTP. Chemicals supplied by the EPA and NTP were from different suppliers/lots, and both were different from those used in ToxCast™ Phase I. Although the EPA and NTP attempt to provide high-quality chemical stocks to collaborators, the purity and composition of impurities can vary by supplier/lot and may affect assay results. The correct identification and tracking of which chemical stocks were used in the experiments would allow better cross-checking with the ToxCast™ and Tox21 data.

Inconsistencies in results reported by Janesick et al. further complicate comparisons. For example, spirodiclofen and zoxamide were both reported to be active in their 3T3-L1 adipogenesis assay at the lowest concentration tested (0.02 μM) (Janesick et al.’s Figure 2A). However, zoxamide was negative at all concentrations in the confirmatory quantitative real-time reverse-transcription polymerase chain reaction analysis for the expression of the differentiation marker genes *Fabp4*, *Fsp27*, and *Lpl*. Spirodiclofen was mostly negative for these markers as well (Figure 2A). Furthermore, the results in the 3T3-L1 adipogenesis assay do not agree with the GAL4-mPPARγ transactivation concentration-response where PPARγ activity in the transactivation assay occurred at 1.31 μM and 12.76 μM for zoxamide and spirodiclofen, respectively, but apparently induced adipogenesis in 3T3-L1 at 0.02 μM for both chemicals—approximately 2 orders of magnitude difference in concentrations (Figures 1A and 2A). In the mBMSC assay, the data were more consistent between adipogenesis and gene induction, but they disagree qualitatively and quantitatively with the 3T3-L1 findings. Thus, it is not clear how one could definitively compare the results from the Janesick et al. study to the ToxCast™ and Tox21 results on an individual assay basis.

Similar to PPARγ, Janesick and colleagues highlighted the discrepancies of their results with the ToxCast™ RXRα and RXRβ results. For RXRβ, the authors pointed out that many apparently selective activators of RXRβ are identified in the Attagene assay despite literature evidence that such selectivity (in the search for receptor subtype-selective ligands) is likely very rare. We agree that this is an unusual finding. We note that the ToxCast™ results, generated with a chimeric GAL4 DNA-binding domain/RXRβ ligand-binding domain, are reproducible. In the interest of transparency, we chose to make these results available despite having no prior reports or basis for understanding with respect to chemical structure. For RXRα, Janesick and colleagues claimed that “triphenyltin, a known PPARγ and RXRα agonist, was not on the Attagene list of RXRα activators (false negative).” The current iCSS Dashboard shows triphenyltin chloride (CAS No. 639-58-7) as active in PPARγ, RXRα, and RXRβ, and triphenyltin hydroxide (CAS No. 76-87-9) as active in PPARγ and RXRβ but not RXRα. In considering all of the ToxCast™ data, it is evident that triphenyltin is highly cytotoxic and invokes cellular stress pathways and cell death across numerous cell types with submicromolar potency. Thus, it can be difficult to capture specific target activity in cell-based assays when general cytotoxicity is occurring at similar concentrations, which appears to be the case for the ToxCast™ RXRα assay. Nonetheless, triphenyltin was still identified as a potent activator of PPARγ and RXR activities in the Attagene assay platform. Although we have not yet developed an integrated model for RXR activity, our current list of most likely RXR agonists is driven by a combination of RXRα and RXRβ activity, and the associated efficacy values for those assays. The EPA can provide these chemicals in a blinded fashion to interested parties for follow-up testing in other assay platforms.

In discussing some of the discrepancies observed in their analysis, Janesick and colleagues speculated as to why concordance was relatively poor for PPARγ and adipogenesis compared with what they deemed to be similar classification models for chemical effects on the androgen receptor and the estrogen receptor. Their reasoning primarily addressed the quality of the input data. An important lesson of the ToxCast™ program is that no individual assay or data point should be considered in isolation or taken as “truth” without consideration of the broader assay and data context, a host of technical and statistical factors derived from experience in working with the data, and both potency and efficacy. The authors appeared to use only potency and ignored efficacy values in selecting compounds despite expected differences in biological response associated with varied efficacy. They also appeared to select chemicals active only in the ATG PPARγ *trans* assay, a GAL4 system containing only a partial PPARγ receptor (ligand-binding domain), and not also in the ATG PPRE *cis* assay, which is responsive to endogenous, full-length PPARγ. While the latter assay is not specific to PPARγ, as the authors correctly point out, in combination with PPARγ *trans* assay it provides higher confidence for additional follow-up. Due to the relatively low dynamic range of the endogenous PPARγ response, interpreting these data can be challenging and go beyond relying on a single potency value.

**Table 1:** Reanalysis of the Janesick et al. chemicals and selected reference chemicals in the 4 ToxCast™/Tox21 assays using *Z*-score

Name (CAS No.)	PPARγ Activity Call^*a*^	Source (Janesick et al. or ToxCast™/Tox21 Reference Compound)^*b*^	No. ToxCast™ Assays with High *Z*-score (total)	No. ToxCast™ Assays with High *Z*-score (agonist)	*Z*-Score for ATG_PPRE_CIS_up assay^*c*^	*Z*-Score for ATG_PPARg_TRANS_up assay^*c*^	*Z*-Score for NVS_NR_hPPARg assay^*c*^	*Z*-Score for TOX21_PPARg_BLA_Agonist_ratio assay^*c*^	*Z*-Score for TOX21_PPARg_BLA_antagonist_ratio assay^*c*^
PD-0333941 (501027-49-2)	agonist	Reference	5	4	15.54	17.15	8.97	18.99	5.84
Farglitazar (196808-45-4)	agonist	Reference	4	4	16.36	18.16	16.48	23.43	2.33
Pioglitazone hydrochloride (112529-15-4)	agonist	Reference	4	4	6.18	10.95	4.70	13.38
Troglitazone (97322-87-7)	agonist	Reference	3	3	9.59	8.56	0.22	12.03
Zoxamide (156052-68-5)	agonist	Table 1	2	1		4.97			6.85
Spirodiclofen (148477-71-8)	agonist	Table 1	3	3	7.05	4.55		5.46
Triphenyltin (76-87-9)	agonist	Table 1, 2	3	2	8.05	6.11	1.08	–3.77	6.70
Pyridaben (96489-71-3)	agonist	Table 2	1	1	4.49	0.64
Triflumizole (68694-11-1)	agonist	Table 1	2	2	3.93	4.48	0.08	–0.02
Quinoxyfen (124495-18-7)	agonist	Table 1, 2	1	1	3.99	2.77		–0.06
Acetochlor (34256-82-1)	antagonist	Table 1	2	1		4.41			3.83
Alachlor (15972-60-8)	antagonist	Table 1	2	1		4.15	–0.66		4.18
Fluazinam (79622-59-6)	antagonist	Table 1, 2	1	1		1.86	3.18		2.07
Fenpyroximate -Z,E (111812-58-9)	inactive	Table 1, 2	2	2	9.74	3.21
Pyraclostrobin (175013-18-0)	inactive	Table 1	3	2	6.39	4.98			5.08
Tebufenpyrad (119168-77-3)	inactive	Table 1, 2	2	2	6.90	3.98			0.99
Dimethenamid (87674-68-8)	inactive	Table 1	1	1		3.79			1.73
Acetamiprid (135410-20-7)	inactive	Table 2	0	0
Asulam (3337-71-1)	inactive	Table 2	0	0
Bisphenol A (80-05-7)	inactive	Table 2	0	0	1.36				0.18
Chlorothalonil (1897-45-6)	inactive	Table 1	0	0			1.79	–4.27	2.10
Cyazofamid (120116-88-3)	inactive	Table 1, 2	0	0	2.07	1.84			0.37
d-cis/trans Allethrin (584-79-2)	inactive	Table 2	0	0	2.23	1.83
Dimethomorph (110488-70-5)	inactive	Table 1	0	0	–0.31
Fenthion (55-38-9)	inactive	Table 2	0	0	1.08	–0.28
Fludioxonil (131341-86-1)	inactive	Table 2	0	0	0.28	-0.16
Flumetsulam (98967-40-9)	inactive	Table 2	0	0
Flusilazole (85509-19-9)	inactive	Table 2	0	0	0.69	0.29
Forchlorfenuron (68157-60-8)	inactive	Table 2	0	0	2.16	–0.02			1.36
Imazalil (35554-44-0)	inactive	Table 2	0	0		0.21
Indoxacarb (173584-44-6)	inactive	Table 1	0	0		0.56		–1.71
Maleic hydrazide (123-33-1)	inactive	Table 2	0	0
Methylene dithiocyanate (6317-18-6)	inactive	Table 2	0	0				–2.01	–0.22
Monocrotophos (6923-22-4)	inactive	Table 2	0	0
Niclosamide (50-65-7)	inactive	Table 1	0	0	0.24				–6.05
PFOS (1763-23-1)	inactive	Table 2	0	0	0.61	0.87	2.15
Prallethrin (23031-36-9)	inactive	Table 2	0	0	2.58	0.52
Prochloraz (67747-09-5)	inactive	Table 1	0	0
Pymetrozine (123312-89-0)	inactive	Table 2	0	0
S-Bioallethrin (28434-00-6)	inactive	Table 1	0	0	2.99	2.35
Tebufenozide (112410-23-8)	inactive	Table 1	0	0	0.04
Tebupirimfos (96182-53-5)	inactive	Table 2	0	0
^***a***^Agonist, antagonist, or inactive call. ^***b***^Source of the agonist, antagonist or inactive call. Table 1 and Table 2 come from Janesick et al. (2016). ToxCast™/Tox21 reference compounds were also included for comparison. ^***c***^Further assay details can be found on the iCSS Dashboard (https://actor.epa.gov/dashboard/) or data download page (https://www.epa.gov/chemical-research/toxicity-forecaster-toxcasttm-data). Note: ATG, Attagene; NVS, Novascreen; TOX21, U.S. Federal Tox21 consortium.									

Apart from needing to consider both potency and efficacy of relevant assays, all assay platforms have limitations and interferences that can result in false positive or false negative results (Thorne et al. 2010). For instance, fluorescence polarization assays such as the Novascreen PPARγ binding assay are prone to interference from fluorescent compounds that emit in an assay-relevant wavelength (Turek-Etienne et al. 2003). Among the statistical considerations, systematic variation can lead to false positive or false negative results when testing on a large scale (e.g., a test with 99% accuracy will have 1 incorrect determination for every 100 compounds tested, and ToxCast™/Tox21 contains thousands of chemicals). In the ToxCast™ data analysis pipeline, active hit calls are largely based on statistical approaches aimed at minimizing false negatives. Whereas this methodology supports overall the application of the data to screening for hazard assessment, the minimization of false negatives on an individual assay basis can have the converse effect of increasing the likelihood of false positives. One of the improvements in the current ToxCast™ data processing pipeline has been the implementation of data quality flags that alert users to potential technical or statistical concerns such as spurious activity or noisy data (Filer et al. 2015). These alerts allow users to tailor their analysis with the appropriate degree of sensitivity and specificity required for their purpose. Although these flags were available to Janesick and colleagues, it appears that they were not utilized in their analyses.

In selecting the chemicals for targeted analysis, Janesick and colleagues used the *Z*-score metric together with potency as a means to separate “true” biological activity (pathway- and target-specific activity) from “false” activity linked to cytotoxicity. Briefly, the *Z*-score is the number of standard deviations that separates the potency for the specified target or pathway from the median potency of a range of cytotoxicity assays (Judson et al. 2015). Janesick and colleagues reported that “on the positive side, applying *Z*-scores, nearly all false negative ToxPi chemicals are lost, or ranked very low,” but “[i]ncredibly, all true positives we identified are also lost”. Our analysis of the same chemicals and *Z*-score data cited by Janesick and colleagues suggested that the authors have incorrectly implemented the calculations (see Table 1). All 6 of the putative agonists reported by Janesick and colleagues had at least 1 ToxCast™/Tox21 assay with a *Z*-score > 3 (3 of the 6 have 2 such assays), meaning that the observed bioactivity was not statistically linked to cytotoxicity. Among these, triphenyltin had *Z*-scores > 3 in the 2 Attagene assays, although the Tox21 PPARγ agonist assay did not show a significant *Z*-score. Whereas the latter result seems at odds with the reported agonist activity for triphenyltin (Harada et al. 2015), consideration of the ToxCast™ data quality flags indicated that the estimated potency needed to be interpreted with caution. Notably, the 4 ToxCast™/Tox21 reference agonists all had multiple assays with *Z*-scores > 3. Among the 3 putative antagonists reported by Janesick and colleagues, all had at least 1 ToxCast™/Tox21 assay with a *Z*-score > 3. Finally, among the 29 putative inactive chemicals reported by Janesick and colleagues, only 4 had at least 1 ToxCast™/Tox21 assay with a *Z*-score > 3. The data for 2 of these 4 chemicals have no data quality flags and deserve follow-up confirmation using orthogonal assays.

Apart from speculating as to the reasons for the observed discrepancies in their analysis, Janesick and colleagues proceeded to express concern on the use of “pre-existing commercial assays”… “designed to identify only the strongest hits in large libraries of structurally similar chemicals (millions or more).” In fact, structurally diverse libraries are screened in lead-generation activities in the pharmaceutical industry. However, there are usually many structural relatives of compounds included in a screening library (along with a diversity of such series), and active series of structurally similar chemicals are statistically unlikely to be missed.

In contrast, we adapted the pharmaceutical strategy to toxicity screening in ToxCast™/Tox21 by working to minimize false negatives through relatively high screening concentrations (100–200 μM in ToxCast™/Tox21 versus 10–20 μM in pharma) and by testing in concentration-response format in most assays (Shukla et al. 2010). One significant difference between pharmaceutical and ToxCast™/Tox21 testing is in the nature of the chemicals undergoing testing. Whereas the pharmaceutical industry typically prescreens their compounds to remove predicted “bad actors,” e.g., aggregators and detergents, the nature of industrial and environmental compounds requires that the ToxCast™/Tox21 libraries include many such chemicals. This increases the likelihood of interference with *in vitro* assays and will always pose a challenge for interpretation of results. For that reason, it is likely that there will always be false positive results from such high-throughput screening assays, which makes the appropriate use of counter-screens, data quality flags, and interpretation of the results all the more important.

Finally, Janesick and colleagues concluded with a recommendation for eliminating the averaging of results across assays in favor of eliminating poorly performing assays. First, we do not average results across assays as a matter of standard practice. Instead, our philosophy is that models should weight assay results as appropriate in the context of the model being developed. Second, poorly performing assays, when an appropriate metric is available for making that determination, or assays with very low (or no) sensitivity have already been eliminated (Huang et al. 2011). Readers should consult our recent estrogen model paper, which demonstrates a variety of approaches for dealing with variable performance across assays, model-based prediction of overall activity, and validation against well-characterized reference chemicals (Judson et al. 2015). Given the lack of complete understanding of the biological relevance of many *in vitro* assays, their technological limitations, and the potential value of the information provided by the assay within the larger ToxCast™/Tox21 data matrix, we have deliberately chosen to keep all assays that could potentially contribute to understanding a compound’s bioactivity. We are a long way from a full understanding of the ToxCast™/Tox21 data generated to date or their potential utility and relevance to modeling the complexity of animal toxicity. Given the current limitations of understanding and the ambitious scope of the ToxCast™ and Tox21 projects in both chemical and biological dimensions, an approach of narrowly applying small slices of the data is likely to be potentially misleading and may miss important chemical effects (i.e., not be health protective).

In summary, the discordance reported by Janesick and colleagues between the ToxCast™ data and the results from their suite of PPARγ and adipogenesis assays is difficult to assess due to a range of methodological, platform, and reagent differences as well as inconsistencies in their assay results. In spite of this, we found a surprising amount of agreement in the overall activity of the chemicals when the results from all relevant assays, cytotoxicity, and data quality flags were considered. This finding is consistent with our previous studies, where integrating the results from multiple orthogonal assays showed greater predictivity than individual assays in isolation (Browne et al. 2015; Judson et al. 2015).

Although we have not yet developed an integrated PPARγ model, we are willing to provide our current pre-publication list of PPARγ modulators as well as chemicals for testing in a blinded fashion in exchange for the raw data and the opportunity to contribute expertise to the data analysis. A hallmark of the ToxCast™ project is the open and transparent sharing of chemicals, data, and expertise as a means to encourage collaboration and strengthen understanding of the potential toxicity of the chemicals. We note that the Blumberg lab used ToxCast™/Tox21 data to prioritize triflumizole as a potential PPARγ ligand and successfully showed that the chemical increased body weight in mice (Li et al. 2012). This analysis was possible only through the public availability of ToxCast™ data. We thank the authors for their interest in analyzing ToxCast™ and Tox21 findings. However, we reiterate that individual ToxCast™/Tox21 assay results provide only 1 piece of a complex puzzle and must be considered within the larger ToxCast™ data context in order to advance understanding of potential human chemical hazards and their mechanisms of activity.
